# Spontaneous Splenic Artery Rupture as the First Symptom of Systemic Amyloidosis

**DOI:** 10.1155/2021/6676407

**Published:** 2021-03-08

**Authors:** Øyvind Bruserud, Tor Henrik Anderson Tvedt, Aymen Bushra Ahmed, Olav Karsten Vintermyr, Tor Hervig, Anne Berit Guttormsen, Håkon Reikvam

**Affiliations:** ^1^Department of Anaesthesia and Intensive Care, Haukeland University Hospital, Bergen, Norway; ^2^Department of Medicine, Haukeland University Hospital, Bergen, Norway; ^3^Department of Pathology, Haukeland University Hospital, Bergen, Norway; ^4^Department of Clinical Science, University of Bergen, Bergen, Norway; ^5^Department of Immunology and Transfusion Medicine, Haugesund Hospital, Norway; ^6^Department of Clinical Medicine, University of Bergen, Bergen, Norway

## Abstract

Spontaneous splenic rupture is a life-threatening condition leading to a rapidly progressing hypovolemic shock due to intra-abdominal blood loss, with a mortality rate of about 10%. Spontaneous splenic rupture can be caused by widely different disorders including acute and chronic infections, neoplastic disorders, and inflammatory noninfectious disorders. In this case report, we present a 67-year-old male patient with hemorrhagic shock caused by an acute bleeding from the splenic artery. The patient was massively transfused with blood products and fluids and underwent laparotomy for hemostatic control and clinical stabilization. Multiorgan involvement by amyloid light-chain amyloidosis (AL-amyloidosis) caused by plasma cell dyscrasia, specifically with infiltration of the spleen artery, was found to be the underlying cause of his life-threatening bleeding. Based on this case, we discuss the features of serious spleen bleeding, massive transfusion therapy in the intensive care setting, and AL-amyloidosis pathophysiology and treatment.

## 1. Introduction

Spontaneous splenic rupture can be caused by several different disorders including acute and chronic infections, neoplastic disorders, and inflammatory noninfectious disorders and has the potential to cause a hemorrhagic life-threatening form of hypovolemic shock [1]. Injuries that penetrate the capsule result in an acute bleeding, while damage to the parenchymal tissue leads to subcapsular hematoma and a delayed rupture. Although splenic rupture is a well-known entity, it is relatively rarely occurring, and only a very few studies have systematically evaluated its pathophysiology and outcome. In this case report, we present a 67-year-old male patient with hemorrhagic shock caused by an acute bleeding from the splenic artery. The patient required massive transfusion therapy and underwent laparotomy for hemostatic control and clinical stabilization. Multiorgan involvement by amyloid light-chain (AL-) amyloidosis was found to be the underlying cause of bleeding.

## 2. Case Report

**Figure 1:**
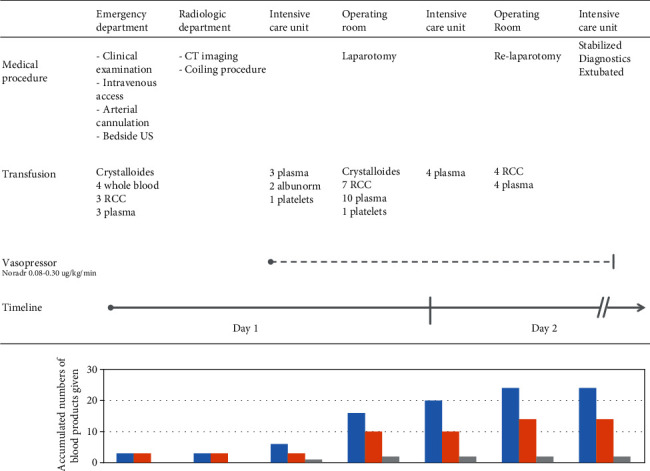
The figure gives an overview over the medical procedures and massive transfusion therapy the patient received for the first two days in hospital. He was initially hemodynamically stabilized by transfusion therapy in the emergency department before the CT imaging and coiling procedure. However, vasopressor was then needed to keep the mean arterial pressure > 60 mmHg and he was massively transfused during laparotomy. The patient finally stabilized the second day in hospital after a relaparotomy to secure hemostasis. The bar chart gives the accumulated number of blood products given; blue bar, plasma; orange bar, red cell concentrates (RCC); and grey bar, platelet concentrates.

A 67-year-old man was admitted to the emergency department (ED) with acute abdominal pain and hypotension. Physical examination revealed blood pressure at 60/30 mmHg, pulse 110 per minute, and a respiratory rate at 40 per minute. He was pale and the abdomen was distended and painful, although without clear peritoneal signs. The patient was suspected of having a hemorrhagic shock caused by an aortic dissection, and resuscitation with fluids and blood products was immediately initiated. The initial laboratory tests are presented in Table 1 and the arterial blood gas analyses in Table 2. Bedside ultrasonography (US) demonstrated a significant amount of free fluid in the abdominal cavity, and a computer tomography (CT) scan revealed an ongoing bleeding from the splenic artery. The patient underwent an emergency endovascular procedure with placement of coils and absorbable gelatin sponge, Spontostan™ (Ethicon, Cincinnati, Ohio), in the splenic artery. However, adequate hemostasis was not achieved, and the patient remained hemodynamically unstable, requiring increased doses of vasopressor (noradrenaline 0.25-0.30 *μ*g/kg/min), while serum lactate was increasing (9.1 mmol/L) and he was continuously resuscitated with blood products (Figure 1).

The patient therefore underwent acute laparotomy revealing massive hemoperitoneum (4.5 litres). Specifically, splenectomy and pancreas resection were considered lifesaving to receive relative hemostasis. The spleen and the liver were found significantly enlarged and hard by intra-abdominal palpation. He was observed in the intensive care unit (ICU) for the next 12 hours with vacuum-assisted closure (VAC) of the abdomen. A relaparotomy was performed the next day to secure hemostasis. He had been massively transfused and received in total four units of whole blood, 13 units of packed red blood cells (RBCs), 24 units of fresh frozen plasma, and two platelet concentrates (Figure 1). The patient stabilized postoperatively and was extubated two days after admittance. In the following days, he developed prerenal kidney failure and liver failure with severe ascites and underwent non-ST-elevation myocardial infarction, all conservatively treated. He was discharged from the ICU after six days.

**Figure 2:**
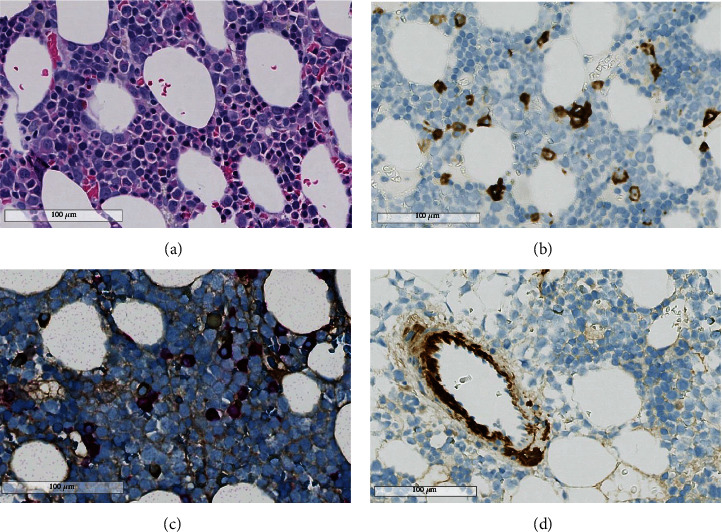
(a) Illustration of representative bone marrow morphology on a formalin-fixed paraffin-embedded hematoxylin-eosin-stained section. (b–d) Immune histochemistry on formalin-fixed paraffin-embedded cut sections using CD138 (b), light chain kappa and lambda (c), and amyloid P (d). There were less than 10% plasma cells (b), and the plasma cells did selective express light-chain lambda (c), and focal deposits of amyloid (d) were noted in vessel walls.

**Figure 3:**
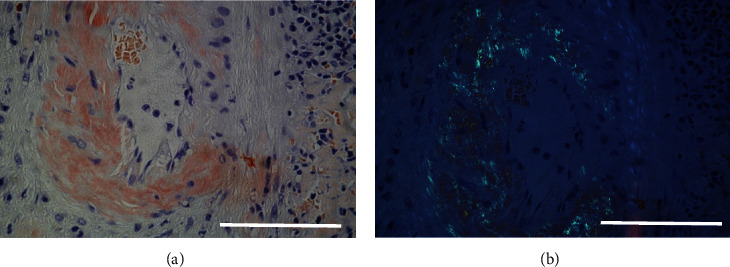
Formalin-fixed paraffin-embedded tissue section from the spleen. (a) Amyloid deposits in an arterial wall using Congo staining. Bar: 100 mm. (b) Green fluorescence due to amyloid deposits in an arterial wall using polarized light on a Congo-stained formalin-fixed paraffin-embedded tissue section from the spleen. Bar: 100 mm.

Based on the findings of hepatosplenomegaly, massive spontaneous hemorrhage, and thrombocytopenia, a hematologic disorder was suspected. Serum analysis revealed increased levels of lambda free light chain (78 mg/L, reference value (RV) 8.3-27.0), while kappa free light chain was normal at 20.1 mg/L (RV 6.7-22.4), resulting in an abnormal serum free light-chain kappa/lambda ratio (0.27 (RV0.31-1.56)). Bone marrow biopsy revealed expanded erythropoiesis and presence of a clonal plasma cell population (5-8% of nucleated cells), staining positive for lambda light chains (Figure 2). Serum electrophoresis and flow cytometry detected a pathological monoclonal plasma cell population expressing lambda light chain (immunophenotype CD45^weakpos^, CD38^pos^, CD138^pos^, CD19^neg^, and CD59^neg^). Congo staining of the splenic tissue revealed areas with amorphic material, appearing red under normal light and green under polarization light (Figure 3). Immunohistochemistry revealed that these deposits, in addition to the deposits in the blood vessels, were positive for amyloid P, amyloid A, and lambda light chains (Figure 3). Mass spectrometry of splenic tissue confirmed the protein in the amyloid deposits to be immunoglobulin lambda light, thereby confirming deposits of AL-amyloid.

Cardiac involvement was found based on significantly elevated levels of serum cardiac markers (NT-proBNP (8619 ng/L, RV < 211 ng/L) and troponin T (223 ng/L, RV < 15 ng/L)), and he was therefore diagnosed with AL-amyloidosis Mayo stage III [2, 3]. Echocardiography revealed a hypokinetic inferior wall, ejection fraction of 65-70%, and moderate aortic stenosis. Moreover, a cardiac magnetic resonance (MR) examination showed a hypertrophic left ventricle with pathologic contrast loading, especially in the septum, due to cardiac amyloidosis.

Our patient received dose-adjusted treatment with cyclophosphamide, dexamethasone, and bortezomib. He improved clinically with disappearance of ascites, leg edema, and improved cardiac function (NYHA stage 0-I). However, no change in serum lambda levels was observed during three courses of chemotherapy. Treatment was therefore changed to daratumumab, a monoclonal antibody that targets CD38 expressed in plasma cells. Three weeks after the first injection of daratumumab, normalization of serum lambda levels was observed. The patient stayed in hospital for 30 days and is currently doing well.

## 3. Discussion

In this case, spontaneous rupture of the spleen artery caused by systemic amyloidosis was found to be the underlying cause of hemorrhagic shock. However, the potential sources of hemorrhage should be identified during the initial evaluation of patients with hemorrhagic shock. Bedside US can be helpful to identify occult sources of bleeding such as a ruptured abdominal aortic aneurysm or a ruptured spleen. Notably, CT imaging should only be performed if the source of bleeding remains uncertain and the patient's condition has been stabilized with initial resuscitation. In cases with severe hemorrhage, the patient is often better served by rapid diagnostic and therapeutic interventions, such as operative exploration, angiography with embolization, or gastrointestinal endoscopy.

The initial management of patients with hemorrhagic shock should focus on restoring the intravascular volume and rapidly localize and control of hemorrhage. Massive-transfusion protocols provide a survival benefit for patients with acute bleeding [4] and mobilize universal donor blood products (e.g., RBCs, plasma, and platelet concentrates) to the patient's bedside in prespecified ratios, together with pharmaceutical adjuncts such as calcium and tranexamic acid. A ratio of red cell concentrates, plasma, and platelet concentrates close to 1 : 1 : 1 is suggested to avoid dilution of coagulation factors and thrombocytopenia [4–8]. Transfusion of whole blood can also be administrated in massive hemorrhage [9]. Laboratory measurements including base deficit, lactate, hemoglobin, international normalized ratio, platelet count, fibrinogen, electrolytes, and thromboelastography (TEG) are crucial for guiding the blood product resuscitation [1].

Amyloidosis is a term referring to a group of complex diseases caused by protein misfolding and aggregation into highly ordered amyloid fibrils depositing in tissues, resulting in progressive organ damage [10]. AL-amyloidosis is a complication of plasma cell dyscrasia, a monoclonal plasma cell population producing a monoclonal antibody or light chains with potential to deposit in several organs. Organ dysfunction is caused by several mechanisms including a direct cytotoxic effect of light chain and bystander proteins, as well as spatial disruption by the amyloid deposits. Cardiac involvement is by far the most significant negative prognostic marker, and the diagnosis can be made using markers of myocardial involvement, cNT-proBNP, and troponin [2].

Although rare, these diseases can also be associated with potentially life-threatening hemorrhage [11, 12]. The pathophysiological mechanisms predisposing for abnormal hemorrhage in patients with systemic amyloidosis are heterogeneous [11]. Amyloid is often associated with acquired coagulation-factor deficiencies [13, 14]. Amyloid fibrils bind and remove factor X from plasma thus inducing a factor X deficiency that is resistant to replacement therapy. Spontaneously elevated international normalized ratio (INR) and activated partial thromboplastin time (aPTT) may indicate factor X deficiency. This acquired factor X deficiency is found in about 1/3 of AL-amyloidosis patients, and splenectomy results in normalization of factor X levels [11]. Isolated deficiency of other coagulation factors, e.g., factor II, V, VII, and IX, has also been described. In addition, abnormal fibrin polymerization and hyperfibrinolysis are also described to be involved in AL-amyloidosis coagulopathy [15, 16]. Platelet dysfunction reflected by decreased aggregability of platelets upon stimulation is found in patients with systemic amyloidosis and may contribute to abnormal hemorrhage [11]. Finally, amyloid deposition in blood vessels and perivascular tissue may lead to amyloid angiopathy with increased vessel fragility and impaired vasoconstriction [17, 18], which was found to be the cause of the splenic artery rupture in our patient.

Spontaneous rupture of the spleen or splenic artery is a rare condition. Although a large number of single case reports have been published, only a very limited number of articles have systematically analyzed cause and treatment outcome after spontaneous spleen rupture. Renzulli et al. [19] reviewed 632 publications reporting a total of 845 patients. In this material, approximately 30% of spontaneous spleen rupture were due to neoplastic disorders, 27% due to infectious disorders, 20% to inflammatory noninfectious disorders, and the remaining cases to mechanical trauma, related to medical treatment, or of unknown etiology. Among neoplastic disorders, hematological malignancies, i.e., non-Hodgkin's lymphomas, acute myeloid leukemia, and myeloproliferative disorders, were the most common causes, although also pancreatic tail tumors with spleen involvement can cause spleen rupture [20]. Mononucleosis was the most common cause among patients with an infectious disorder, reported in 102 of 137 cases (74%). Approximately 85% of patients required surgical treatment, and the reported mortality was 12%. Age and neoplastic disorders conferred a higher probability of death.

However, the primary treatment goal of AL-amyloidosis is to eliminate the monoclonal plasma cell clone producing the pathological light chain. Hence, AL-amyloidosis applies similar treatment protocols as for multiple myeloma, with the goal of a rapid suppression of the amyloidogenic organ toxic light chains. Particularly, the proteasome inhibitor bortezomib that ensures a rapid reduction in plasma cell light-chain production is often incorporated into treatment protocols [10]. Nevertheless, patients with amyloidosis are often too fragile, due to age and to organ involvement, to tolerate the most intensive approach which includes high-dose chemotherapy and autologous hematopoietic stem cell transplantation [10], and patient-adapted risk assessment is required to reduce the treatment-related mortality.

## 4. Conclusion

Spontaneous splenic rupture is a rare condition leading to a rapidly progressing life-threatening hypovolemic shock due to intra-abdominal blood loss and can be caused by disorders such as acute and chronic infections, neoplastic disorders, and inflammatory noninfectious disorders. Although rare, plasma cell dyscrasia causing amyloid light-chain amyloidosis also has the potential to cause spontaneous splenic rupture and life-threatening bleeding. However, early recognition of hemorrhagic shock and prompt action to stop the bleeding and restore the patient's intravascular volume and oxygen-carrying capacity are lifesaving.

## Figures and Tables

**Table 1:** The table gives the laboratory tests at the emergency department.

	Normal range	Value
Hemoglobin	13.4–17.0 g/dL	9.2
Leucocyte count	4.3–10.7 × 10^9^/L	17.5
Platelet count	145–348 × 10^9^/L	50
Creatinine	45-90 *μ*mol/L	170
Albumin	39-48 g/L	34
Ionized calcium	1.13-1.28 mmol/L	1.20
Lactate dehydrogenase	105-205 U/L	441
C-reactive protein	<5 mg/L	24
INR	0.8-1.2	1.3
APTT	30-44 s	57
Glucose	4.0-6.0 mmol/L	9.0
ALAT	10-70 U/L	134
ASAT	15-45 U/L	207
ALP	35-105 U/L	329
GT	15-115 U/L	386
Bilirubin	<19 *μ*mol/L	25
Troponin T	<15 ng/L	175

Abbreviations: ALAT: alanine aminotransferase; ALP: alkali phosphatase; APTT: activated partial thromboplastin time; ASAT: aspartate aminotransferase; INR: international normalized ratio; GT: gamma-glutamyltransferase.

**Table 2:** Blood gas analysis in the emergency department.

	Normal range	Value
pH	7.36-7.44	7.10
pCO_2_	4.5-6.1 kPa	3.8
pO_2_	>9.2 kPa	31.4
Anion gap	7-12 mmol/L	25.8
Hemoglobin	13.4–17.0 g/dL	9.9
Lactate	0.4-1.3 mmol/L	13.3
Glucose	4.0-6.0 mmol/L	9.0
Sodium	137-145 mmol/L	140
Potassium	3.4-4.8 mmol/L	4.1
Clor	102-110 mmol/L	110
Ionized		
Calcium	1.13-1.28 mmol/L	1.20
